# Population pharmacokinetic characteristics of cemiplimab in patients with advanced malignancies

**DOI:** 10.1007/s10928-021-09739-y

**Published:** 2021-03-16

**Authors:** Feng Yang, Anne J. Paccaly, Ronda K. Rippley, John D. Davis, A. Thomas DiCioccio

**Affiliations:** 1 Regeneron Pharmaceuticals, Inc, 777 Old Saw Mill River Rd, Tarrytown, NY USA; 2 Present Address: Alexion Pharmaceuticals, 121 Seaport Blvd, Boston, MA 02210 USA; 3 Present Address: Constellation Pharmaceuticals, 215 First St UNIT 200, Cambridge, MA 02142 USA

**Keywords:** Population modeling and simulations, Cemiplimab, Fixed dose selection, Covariates, Time-varying clearance

## Abstract

Cemiplimab, a human monoclonal antibody targeting programmed cell death-1 (PD-1) receptor, demonstrated antitumor activity in patients with advanced malignancies and a safety profile comparable to other anti–PD-1 therapies. This population pharmacokinetics (PopPK) analysis of cemiplimab included 11,178 pharmacokinetics (PK) observations from 548 patients pooled from a first-in-human study (Study 1423; NCT02383212) in advanced malignancies and a Phase 2 study (Study 1540; NCT02760498) in advanced cutaneous squamous cell carcinoma (CSCC). Most patients (80.3%) received cemiplimab 3 mg/kg every 2 weeks (Q2W) intravenously (IV). A PopPK model was developed by evaluating two-compartment linear models with an empirical non-linear function describing time-varying change in cemiplimab clearance and covariates that improved goodness-of-fit. PopPK simulations were used to describe cemiplimab exposure generated by a fixed 350 mg every 3 weeks (Q3W) IV dose regimen. PopPK modeling showed that a two-compartment model with zero-order IV infusion rate and first-order elimination rate well described individual concentrations of cemiplimab. Although several covariates, including baseline body weight and albumin concentrations, had a modest impact on cemiplimab exposure, the magnitude of influence was within the typical observed PK variability of approximately 30%. Based on PopPK simulation results, the 350 mg Q3W dose regimen was selected for further studies in advanced malignancies, including advanced CSCC. Similarity in observed cemiplimab exposure at the fixed 350 mg Q3W and the weight-based 3 mg/kg Q2W dose regimens confirmed this fixed dose selection. A robust PopPK model was developed to describe cemiplimab concentrations and supported use of the fixed 350 mg Q3W IV dose regimen.

**Supplementary Information:**

The online version of this article (10.1007/s10928-021-09739-y) contains supplementary material, which is available to authorized users.

## Introduction

Cemiplimab, a high-affinity, human, hinge-stabilized immunoglobulin G4 (IgG4) monoclonal antibody to the programmed cell death-1 (PD-1) receptor, can potently block the interactions of PD-1 with programmed death-ligand 1 and 2 (PD-L1 and PD-L2) [1]. In first-in-human (Study 1423; NCT02383212) and Phase 2 (Study 1540; NCT02760498) studies, cemiplimab has demonstrated antitumor activity, durable responses, and a safety profile similar to those described for other anti–PD-1 therapies in patients with advanced malignancies, including metastatic cutaneous squamous cell carcinoma (mCSCC) or locally advanced cutaneous squamous cell carcinoma (laCSCC), collectively referred to as advanced CSCC [2]. Cemiplimab (cemiplimab-rwlc in the US) is approved for the treatment of patients with advanced CSCC who are not candidates for curative surgery or curative radiation. It is also approved in the US for patients with locally advanced and metastatic basal cell carcinoma (BCC), post hedgehog inhibitors (HHIs) or for whom HHIs are not appropriate. Cemiplimab-rwlc is also approved for the first-line treatment of patients with advanced non-small cell lung cancer (NSCLC) whose tumors have high PD-L1 expression (tumor proportion score ≥50%) and no epidermal growth factor receptor, anaplastic lymphoma kinase or ROS1 aberrations, for patients with metastatic or locally advanced tumors that are not candidates for surgical resection or definitive chemoradiation. [3, 4].

As a human monoclonal antibody directed against the cell membrane target, PD-1, cemiplimab is expected to present a saturable, target-mediated elimination pathway leading to non-linear pharmacokinetics (PK) at low concentrations [5]. Observations from Studies 1423 and 1540 show that the PK of cemiplimab was generally linear and dose proportional after the first dose over a dose range of 1–10 mg/kg administered intravenously (IV) every 2 weeks (Q2W) [3]. The observed linearity of PK indicates systemic saturation of the underlying target-mediated pathways at the concentrations evaluated. A weight-based 3 mg/kg Q2W regimen was initially studied in the Phase 2 trial to ensure maximum therapeutic effect at saturation.

To reduce treatment burden on patients, caregivers, and healthcare systems, as well as to better align with dosing intervals of chemotherapies, a fixed dose of cemiplimab with a longer dosing interval was considered. To select a more preferable dosing regimen, a robust population PK (PopPK) model was developed to characterize the PK parameters and the post hoc concentration profiles of cemiplimab in patients with advanced malignancies, including a subset of patients with advanced CSCC, at different dosing regimens. These post hoc simulation results showed that a fixed 350 mg every-3-week (Q3W) dose regimen was expected to generate similar cemiplimab exposure to that observed with the initially studied weight-based regimen of 3 mg/kg Q2W.

## Methods

### Software

PopPK analysis was conducted by non-linear mixed-effects modeling using NONMEM® (7.4, ICON Development Solutions, Ellicott City, Maryland, USA). NONMEM® was accessed through PsN (4.6.0, Uppsala University, Sweden) and run on a Linux high-performance cluster. Tables and figures were prepared with R version 3.3.1 or above (http://www.r-project.org). PsN 4.6.0 and Xpose 4.5.3 (Uppsala University, Sweden) were used as supportive software for NONMEM®. In addition, R package ‘mrgsolve’ (0.8.0 or above, Metrum Research Group LLC, CT) was used for simulation.

### Observations for model building

The cemiplimab concentrations used for cemiplimab PK modeling were based on cross-study pooling from Study 1423 in patients with advanced malignancies and Study 1540 in patients with advanced CSCC. All patients included in this analysis received cemiplimab as 30-min IV infusions of either a weight-based (1, 3, or 10 mg/kg Q2W, or 3 mg/kg Q3W) or a fixed (200 mg Q2W or 350 mg Q3W) regimen. The duration of treatment was scheduled to be up to 48 weeks in Study 1423 and up to 96 weeks in Study 1540, or until the patient had unacceptable toxicity or confirmed disease progression. In Study 1423, PK observations were obtained through serial sampling during the first cycle prior to and at the end of the 30-min infusion, 1, 4, 8, 24, 48, and 72 h after the first infusion as well as day 8 for both Q2W and Q3W groups, and sparse sampling at trough and/or the end of infusion during cycles 2–6; anti-cemiplimab antibody observations were collected pre-infusion on day 1 of cycles 1, 2, and 4. In Study 1540, PK observations were obtained through sparse sampling at trough and/or the end of infusion on days 1, 15, 29, and 43 of cycle 1, on day 1 of cycles 2–6, 7, 9, and 11, and either at the end of study visit or at the follow-up visit. Anti-drug antibody observations were collected prior to treatment on day 1 of cycles 1, 3, 5, 7, and 11. The last sample collection date for cemiplimab concentration was September 6, 2017 for Study 1423 and January 18, 2018 for Study 1540. The studies were ongoing at the time of last sample collection.

### Data exclusion and BLQ records handling

Data excluded from PopPK analysis were drug concentration observations prior to the first dose of cemiplimab; drug concentration observations below the limit of quantification (BLQ); and outlier observations identified using initial inspection of raw observations, inspection of outputs from the base model, and inspection of diagnostic plots.

In particular, identification of outliers by inspection of raw observations is described here. Concentration–time profile plots of all observations were generated with a non-parametric locally-weighted smoother line. Observations discordant with the bulk of the data within the same time interval and dose group were identified as outliers. In addition, the ratio of the concentration data collected at the end of infusion (C_eoi_) and pre-infusion were calculated; any ratios below 1.0, i.e., minimum concentration (C_trough_) > C_eoi_, were marked as outliers. Concentrations that exceeded five times the average concentrations at specified time points in the related population were marked as outliers.

Using a box-plot approach, potential extreme outliers were defined as points that lie outside the interval $\left[ {q_{1} - 3 \cdot IqR q_{3} + 3 \cdot IqR} \right]$, where q_1_ and q_3_ correspond to the first and third quartiles of the observations, respectively, and the interquartile range (IqR) was defined as $IqR = q_{3} - q_{1}$.

Observations were also classified as outliers using the population conditional weighted residuals (CWRES) and individual weighted residuals (IWRES) during the base model development. Observations with |CWRES|> 5 or |IWRES|> 5 were considered outliers.

By default, BLQ records were handled by flagging for exclusion. When the percentage of post-dose BLQ observations was significant (accounting for ≥ 5% of the sampling observations), a likelihood-based approach (M3) was implemented for BLQ observations. If retaining these BLQ values in the dataset led to convergence problems, failure of the $COV step, or key parameter estimates differing more than 15%, these observations were excluded from the analysis. When the percentage of post-dose BLQ observations accounted for < 5% of the sampling observations, a sensitivity analysis was performed based on a dataset that included these BLQ observations using the M5 method, i.e., replacing BLQ with LLOQ/2 with LLOQ denoting the lower limit of quantification.

The influence of the outliers or BLQ records identified above was evaluated in a sensitivity analysis by comparing estimates of key model parameters, such as clearance (CL) and central volume of distribution (V_2_), from the final model, fitted on data with and without these concentration data.

### Missing data and imputations

For time-variant covariates, partially missing data were imputed using last observation carried forward. For a baseline continuous covariate that was missing at all visits, median value of the covariates across all patients was used; for categorical missing covariates, the value of the most frequent category was used.

### PopPK model development

**Fig. 1:**
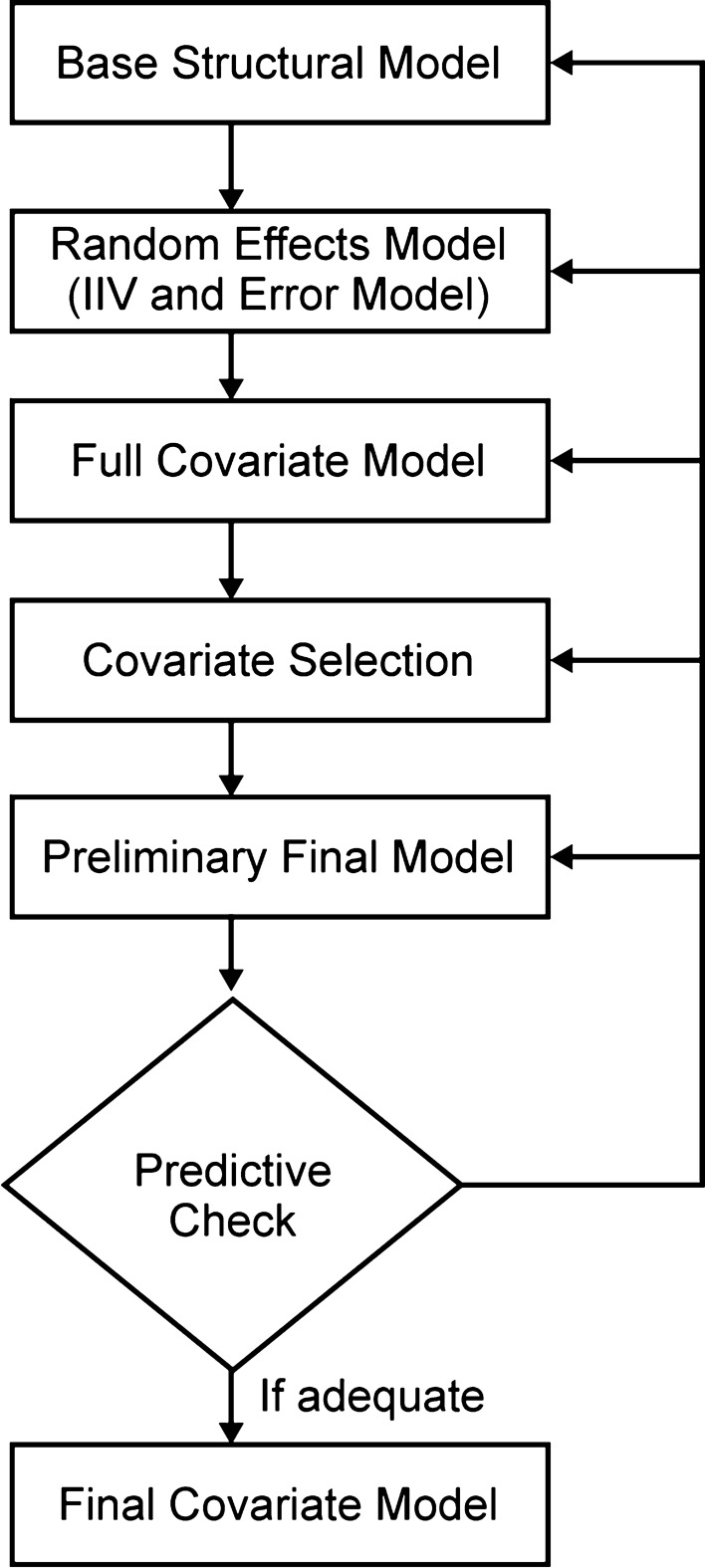
Overview of PopPK analysis. IIV, inter-individual variability; PopPK, population pharmacokinetics

The PopPK model was developed in three stages (Fig. 1).

**Fig. 2:**
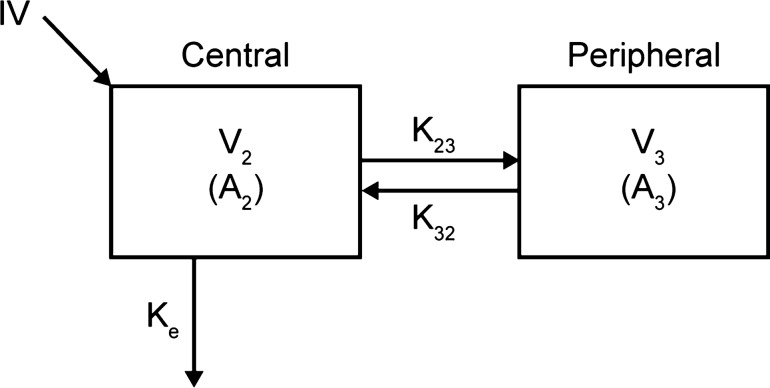
Structural representation of a two-compartment model with linear elimination for IV administration. Plasma clearance was derived from k_e_ × V_2_; inter–compartmental clearance between the central and peripheral compartments (Q) was derived from k_23_ and k_32_. A_2_, the amount of cemiplimab in the central compartment with a volume V_2_; A_3_, the amount of cemiplimab in the peripheral compartment with a volume V_3_; IV, intravenous; k_23_, k_32_, inter-compartmental rate constants; k_e_, elimination rate constant; V_2_, volumes of distribution (central compartment); V_3_, volume of distribution (peripheral compartment)

At stage one, a base model describing the PK of cemiplimab without covariate considerations was created (Fig. 2). The base model development included a structural model, a residual error model, and an inter-individual variability (IIV) model. Various base model structures were assessed, including standard two-compartment PopPK models and parallel constructs of linear and non-linear (Michaelis–Menten) elimination structures. In addition, this study assessed the ability of an empirical non-linear function that enabled CL to monotonically decline over time [6] to improve description of cemiplimab concentrations in serum.

The structural model was parameterized in terms of CL, V_2_, peripheral volume of distribution (V_3_), and inter-compartmental clearance (Q), with log-normal random-effect distributions. In the model with both parallel linear and non-linear elimination, V_max_ (maximum rate in non-linear elimination) and K_m_ (Michaelis–Menten constant) were introduced. The representation in Fig. 2 can be mathematically expressed as the following equations:

$$\frac{{\ {dA_{2} }}}{dt} = -k_{e} A_{2} {-}k_{23} A_{2} + k_{32} A_{3} - \frac{{V_{max} *\frac{{A_{2} }}{{V_{2} }}}}{{k_{m} + \frac{{A_{2} }}{{V_{2} }}}}$$

$$\frac{{dA_{3} }}{dt} = k_{23} A_{2} {-} k_{32} A_{3}$$

A_2_, the amount of cemiplimab in the central compartment with a volume V_2_; A_3_, the amount of cemiplimab in the peripheral compartment with a volume V_3_; k_23_, k_32_, inter-compartmental rate constants; k_e_, elimination rate constant.

Various combinations were used of exponential between-subject variability on CL, Q, V_2_ and V_3_, as well as E_max_ and T_50_, with or without off-diagonal correlation.

Residual variability, a composite measure of assay error, dose/sample time collection errors, model misspecification, and any other unexplained variability within a patient, was described using the following error model,

$$Y = F + F \times ERR\left( 1 \right) + ERR\left( 2 \right)$$

where *Y* denotes the observed concentration; *F* denotes the corresponding predicted concentration based on the PopPK model; *ERR(1)* and *ERR(2)* denote the proportional and additive residual random variables, respectively, and were assumed to have a normal distribution with a zero mean and variance. Log-transformation of the error model was applied.

At stage two, a full covariate model incorporating all pre-specified covariate parameters was developed. To achieve unbiased estimation of covariate effects, two full models (LN101 and LN102) were used to pre-select the potential pairs of covariate-parameter. In particular, LN101 was used to select covariates that may have an impact on CL parameters (CL and Q) and volumes of distribution (V_2_ and V_3_) with 42 pairs of covariate-parameter to be filtered. Model LN102 was used to select covariates that may have an impact on E_max_ and T_50_ in the sigmoid E_max_ term with 42 pairs of covariate-parameter to be filtered. An effect threshold (absolute effect size > 0.1) relative to reference values was used to filter the potential covariates prior to forward addition and backward elimination; both baseline and certain time-variant covariates were assessed. Due to the time-variant CL observed in the preliminary PopPK analyses, time-variant body weight, albumin concentration in serum, and lactate dehydrogenase level were assessed either in a post hoc manner or in the covariate development analysis. Covariates assessed as only baseline parameters were sex, age, race, body mass index (BMI), body surface area, ethnicity (Hispanic or Latino vs non-Hispanic or Latino), country, creatinine concentration, creatinine CL, alanine aminotransferase concentration, aspartate aminotransferase concentration, alkaline phosphatase concentration, total bilirubin concentration, tumor type (CSCC, mCSCC vs laCSCC, and other tumor types), disease and patient characteristics (metastatic vs locally advanced disease, Eastern Cooperative Oncology Group [ECOG] performance status), treatment (monotherapy vs combination therapy), study cohort (dose escalation vs expansion), biomarkers (baseline IgG), and anti-drug antibody status (positive vs negative). The variance of PK parameters was introduced in four model parameters, a shared IIV on CL and Q, a shared IIV on V_2_ and V_3_, as well as IIV on E_max_ and T_50_.

The resulting multivariate model was further processed by standard procedures of forward addition and backward elimination. In forward addition, covariates that contributed ≥ 6.63 change in the minimum objective function value (MOFV) were considered statistically significant and the covariate with the smallest *P*-value was included as the next base model. In backwards elimination, the most non-significant covariate (highest *P-*value > 0.001) that contributed to a < 10.83 change in the MOFV value when removed from the model was eliminated.

At stage three, a final PopPK model retaining covariates that improved goodness-of-fit was established using bootstrap methodology and visual predictive checks (VPCs).

### PopPK simulations and observed data to inform fixed dose selection of cemiplimab

The final PopPK model was used to simulate post hoc concentration–time profiles and calculate the corresponding exposure metrics, using individual predicted model parameters in the analysis population (N = 548).

Simulation of the fixed 350 mg Q3W dose regimen included 2000 patients (mean body weight 75 kg, mean age 60 years, mean BMI 26.5 kg/m^2^, mean albumin 38 g/L, mean IgG level 9.7 g/L, and mean alanine transaminase (ALT) 21 IU/L). If there were two highly correlated covariates (such as body weight and BMI), only one (body weight) was used in the simulation.

The simulation results were used to verify similar cemiplimab exposure to the weight-based 3 mg/kg Q2W dose regimen observed in patients with advanced malignancies. Observed concentrations of cemiplimab were used to confirm the fixed dose regimen selected based on the PopPK modeling and simulations.

## Results

### Analysis set

A total of 11,909 cemiplimab concentration observations from 549 patients with advanced malignancies were used. A total of 731 (6%) observations were excluded based on pre-specified criteria, including 15 observations (< 1% of the total) with BLQ concentration values, many of which were identified as outliers. A total of 51 observations from 34 patients were identified as outliers during the model development using the |CWRES|> 5 or |IWRES|> 5 criteria, including volatile drug concentration observations from one patient which caused instability of the PopPK model. The number of missing continuous and categorical covariates was limited and did not exceed 10% of the overall population studied. A sensitivity analysis was performed to evaluate the influence of these excluded observations by comparing estimates of the key model parameters from the base model fits with and without excluded observations. Including the excluded observations prevented the model from converging, justifying exclusion of such observations from the PopPK analyses. The final PK analysis set included 11,178 cemiplimab concentration observations from 548 patients with solid tumors, including 2266 observations from 178 patients with advanced CSCC (Table 1).

**Table 1:** Patients and cemiplimab concentration data included in the PopPK analysis

Dosing regimen	Overall analysis set		Advanced CSCC analysis set	
	Number of patients	Number of data	Number of patients	Number of data
Study 1423*				
1 mg/kg IV Q2W	27	894	1	54
3 mg/kg IV Q2W	331	7710	25	734
10 mg/kg IV Q2W	6	188	–	–
200 mg IV Q2W	20	672	–	–
3 mg/kg IV Q3W	12	236	–	–
*Study 1540*^†^				
3 mg/kg IV Q2W	109	1264	109	1264
350 mg Q3W	43	214	43	214
Overall	548	11,178	178	2266

*CSCC* cutaneous squamous cell carcinoma, *IV* intravenous, *PD-1* programmed cell death-1 receptor, *PopPK* population pharmacokinetics, *Q2W* every 2 weeks, *Q3W* every 3 weeks

*Study 1423 included patients with advanced malignancies (solid tumors) that were incurable and had failed to respond to, or showed tumor progression, despite standard therapy, or patients who were not candidates for standard therapy, or for whom no available therapy was expected to convey clinical benefit, or for whom PD-1 blockade had been shown to be at least equivalent to standard of care

^†^Study 1540 included patients with metastatic CSCC or locally advanced CSCC

### Baseline characteristics

Demographic characteristics and relevant covariates for the PK analysis population at baseline are summarized in Table 2. Among 548 patients with advanced malignancies included in the overall analysis, median age was 65 (range 27–96) years, 60.4% were male, 90.9% were white, and median weight was 76.2 (range 30.9–172) kg.

**Table 2:** Baseline characteristics

Characteristics	Overall analysis population (n = 548)	Advanced CSCC analysis population (n = 178)
Median age, years (range)	65 (27–96)	72 (38–96)
Male, n (%)	331 (60.4)	149 (83.7)
*Race*		
White	498 (90.9)	172 (96.6)
Black	20 (3.6)	1 (0.6)
Asian	9 (1.6)	2 (1.1)
Other*	21 (3.8)	3 (1.7)
Median weight, kg (range)	76.2 (30.9–172)	80.0 (46.4–172)
Median height, cm (range)	170 (140–199)	174 (140–190)
Median body mass index, kg/m^2^ (range)	26.5 (14.8–56.3)	26.7 (17.2–55.5)
Median creatinine concentration, μmol/L (range)	76.0 (33.6–201)	79.6 (46.0–201)
Median creatinine clearance, mL/min (range)	86.7 (24.9–419)	76.7 (27.7–217)
Median albumin concentration, g/L (range)	38.0 (22.0–48.0)	39.0 (26.0–48.0)
Median IgG concentration, g/L (range)	9.65 (1.29–27.9)	10.1 (3.50–21.6)
Median alanine aminotransferase concentration, IU/L (range)	20.0 (5.00–196)	16.0 (6.00–92.0)

*CSCC* cutaneous squamous cell carcinoma, *IgG* immunoglobulin G, *IU* international unit

*Includes other identified races, races unknown, or not reported

### PopPK model

A two-compartment model with zero-order IV infusion rate, first-order elimination rate, and a time-varying decrease in CL effectively described the concentrations of cemiplimab in serum.

### Base model

A stable and parsimonious base model was developed to describe cemiplimab concentration–time data in patients with advanced malignancies, without considering covariate effects. Models incorporating a Michaelis–Menten elimination term did not improve goodness-of-fit compared with the corresponding linear models. Inclusion of a sigmoid-E_max_ functional form [6], ${\text{exp}}(\frac{{{\text{Emax}}\cdot{\text{T}}^{{\upgamma }} }}{{{\text{T}}50^{{\upgamma }} + {\text{T}}^{{\upgamma }} }}$), which describes the time-varying change in cemiplimab CL, significantly improved the model fit of the two-compartment linear model. On average, CL decreased by 35.9% over time compared with baseline CL (from 0.326 to 0.208 L/day within 16 weeks of treatment). Limited observations indicated a higher decrease in CL in treatment responders versus non-responders under repeated cemiplimab treatment (Supplementary Fig. 1). However, this decrease in CL was not considered clinically meaningful due to limited impact (< 20%; within the typical observed PK variability of approximately 30%) on the resulting exposure (minimum concentration at steady state [C_trough,ss_] and area under the curve over 6 weeks at steady state [AUC_6wks,ss_]) with or without time-variant decrease in CL and the flat exposure–response relationships for clinical efficacy in patients with CSCC and for safety over a wide range of tested doses studied in patients with solid tumors.

Testing of IIV on PK parameters led to the estimation of a shared IIV on CL and Q and a shared IIV on V_2_ and V_3_, as well as the covariance between these two. This covariance structure reduced the over-parameterization and effectively characterized apparent correlations between the parameters with limited impact on objective function value (OFV). It was also consistent with the principle of allometric scaling. In the final covariance structure, inter-subject variabilities of E_max_ and T_50_ were also used and considered as appropriate with acceptable quality criteria.

Subsequently, exploration of various residual variability models led to the selection of the additive and proportional residual error on the log-scale model, which best described the residual variability.

### Covariate model

**Fig. 3:**
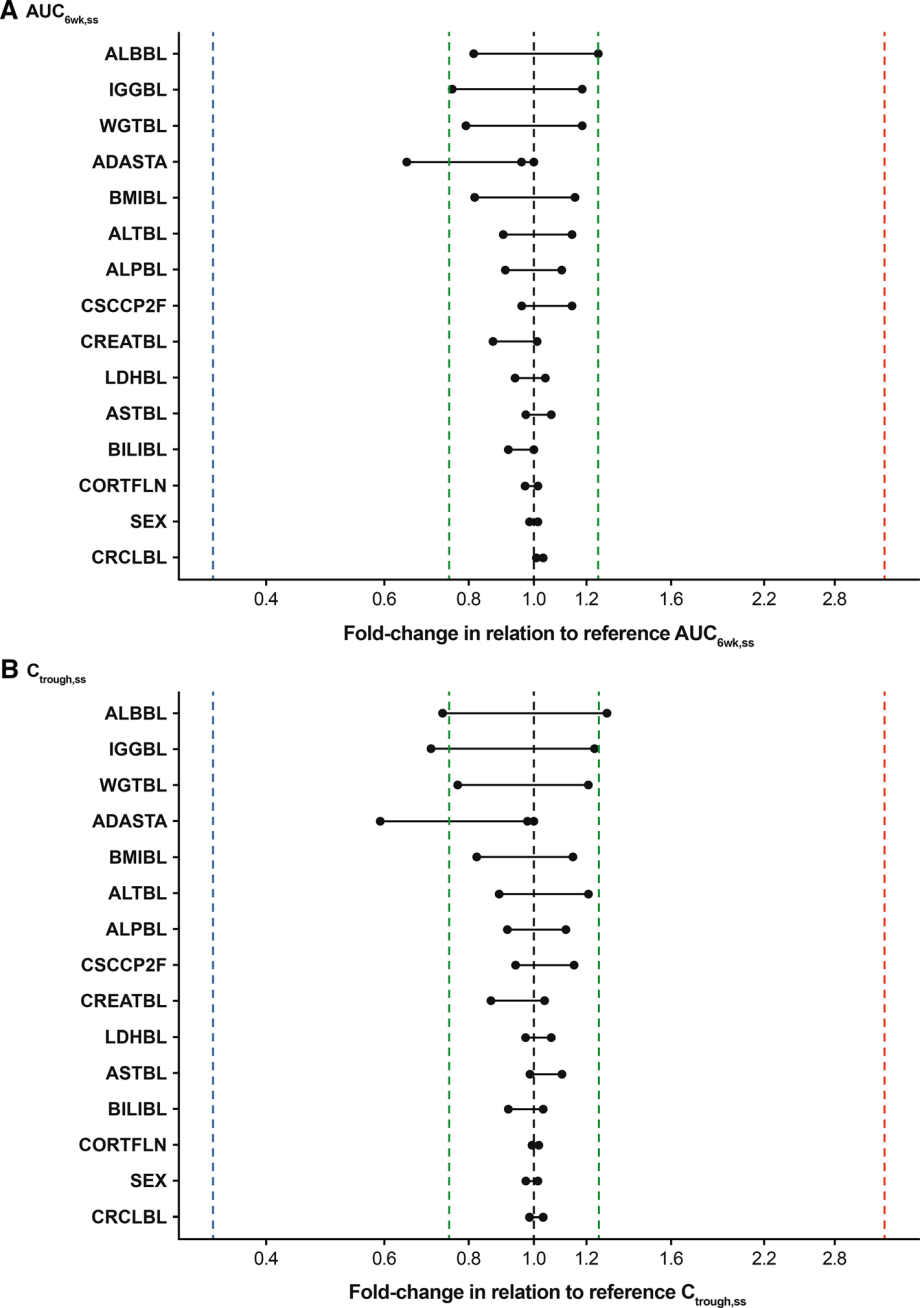
Tornado plots of post hoc steady-state AUC_6wk,ss_ and C_trough,ss_ by relevant covariates at 3 mg/kg Q2W. Dashed black lines represent the steady state median of AUC_6wk,ss_ or C_trough,ss_ at 3 mg/kg Q2W for a typical patient. Solid black lines represent relevant covariates, continuous variables or categorical variables. Black dots represent the relative exposure in sub-populations (either the top 90% percentile or bottom 10% of the relevant covariates) for continuous variables, or sub-populations indicated by categorical variables. The length of the bar from the black dashed line represents the fold change in relation to the reference exposure at 3 mg/kg Q2W. The blue and red lines represent the median exposures at 1 mg/kg Q2W and 10 mg/kg Q2W, respectively. The green lines represent 75% or 125% of the reference exposure. ADASTA, anti-drug antibody status; ALBBL, albumin concentration at baseline; ALPBL, alkaline phosphatase concentration at baseline; ALTBL, alanine aminotransferase concentration at baseline; ASTBL, aspartate aminotransferase concentration at baseline; AUC_6wk,ss_, area under curve over 6 weeks at steady state; BILIBL, total bilirubin concentration at baseline; BMIBL, body mass index at baseline; CORTFLN, corticosteroid (yes or no); CRCLBL, creatinine clearance at baseline; CREATBL, creatinine concentration at baseline; CSCCP2F, cutaneous squamous cell carcinoma (yes or no); C_trough_,_ss_ minimum concentration at steady state; IGGBL, immunoglobulin G concentration at baseline; LDHBL, lactate dehydrogenase concentration at baseline; Q2W, every 2 weeks; WGTBL, body weight at baseline

A full-model approach (LN101 and LN102) was used to pre-select the potential pairs of covariate-parameter. Then covariate analysis with forward addition and backward elimination was conducted to further improve the PopPK model. Covariates that significantly improved the model (*P* < 0.01) were baseline body weight, BMI, albumin concentration in serum, IgG concentration in serum, ALT concentration in serum, and race (Fig. 3). However, due to the limited impact (< 20%) of these covariates on cemiplimab exposure and considering the flat exposure–response relationship for both clinical efficacy and safety, the effects of these covariates did not appear to be clinically meaningful. Tumor type was assessed in the stepwise covariate analysis but did not meet the covariate inclusion criteria for the final PopPK model. Tumor type did not have a clinically meaningful effect on cemiplimab concentration, indicating that cemiplimab PK was similar across tumor types.

### Final PopPK model

The final PopPK model was as follows:

$$CL_{i} = CL_{Base, REF } \cdot \exp \left( {\frac{{Emax_{i} * T^{y} }}{{T50_{i}^{y} + T^{y} }}} \right) \cdot \left( {\frac{{WGTBL_{i} }}{{WGTBL_{REF} }}} \right)^{WGTBL\_ON\_CLQ} \cdot {\text{exp}}(n_{i} ) \cdot \left( {\frac{{ALBBL_{i} }}{{ALBBL_{REF} }}} \right)^{ALBBL\_ON\_CLQ } \cdot \left( {\frac{{IGGBL_{i} }}{{IGGBL_{REF} }}} \right)^{IGGBL\_ON\_CLQ } \cdot \left( {\frac{{ALTBL_{i} }}{{ALTBL_{REF} }}} \right)^{ALTBL\_ON\_CLQ }$$

$$Q_{i} = Q_{REF} \cdot \left( {\frac{{WGTBL_{i} }}{{WGTBL_{REF} }}} \right)^{WGTBL\_ON\_CLQ} \cdot {\text{exp}}(n_{i} ) \cdot \left( {\frac{{ALBBL_{i} }}{{ALBBL_{REF} }}} \right)^{ALBBL\_ON\_CLQ } \cdot \left( {\frac{{IGGBL_{i} }}{{IGGBL_{REF} }}} \right)^{IGGBL\_ON\_CLQ } \cdot \left( {\frac{{ALTBL_{i} }}{{ALTBL_{REF} }}} \right)^{ALTBL\_ON\_CLQ }$$

$$V2_{i} = V2_{REF } \cdot \left( {\frac{{WGTBL_{i} }}{{WGTBL_{REF} }}} \right)^{WGTBL\_ON\_VSS} \cdot \left( {\frac{{BMIBL_{i} }}{{BMIBL_{REF} }}} \right)^{BMIBL\_ON\_VSS} \cdot {\text{exp}}(n_{i} )$$

$$V3_{i} = V3_{REF } \cdot \left( {\frac{{WGTBL_{i} }}{{WGTBL_{REF} }}} \right)^{WGTBL\_ON\_VSS} \cdot \left( {\frac{{BMIBL_{i} }}{{BMIBL_{REF} }}} \right)^{BMIBL\_ON\_VSS} \cdot {\text{exp}}(n_{i} )$$

$$T50_{i} = T50_{REF} \cdot \left( {BLK} \right)^{BLK\_ON\_T50} \cdot {\text{exp}}(n_{i} )$$

$$EMAX_{i} = EMAX_{REF} \cdot {\text{exp}}(n_{i} )$$

This model used typical PK parameter estimates (CL_base,REF_, Q_REF_, V_2,REF_, V_3,REF_, T_50,REF_, and E_MAX,REF_) and median covariate values to assess the covariate effects of baseline body weight, albumin, IgG, ALT, BMI, and race (WGTBL_ON_CLQ, ALBBL_ON_CLQ, IGGBL_ON_CLQ, ALTBL_ON_CLQ, WGTBL_ON_VSS, BMIBL_ON_VSS, and BLK_ON_T50). PopPK parameters obtained from the final model are presented in Table 3.

**Table 3:** PopPK parameters from the final PopPK model

Parameter	Description of PopPK parameters	Estimate (relative standard error) of PopPK parameters obtained from the final PopPK model, (%)
TVCL	Clearance	0.290 L/day (2.38)
TVV_2_	Central volume of distribution	3.32 L (1.10)
TVQ	Inter-compartmental clearance	0.638 L/day (4.93)
TVV_3_	Peripheral volume of distribution	1.65 L (3.37)
RUVCV	Proportional error	0.188 (0.319)
RUVSD	Additive error	1.48 mg/L (4.72)
E_max_	Maximum effect in sigmoid model	− 0.410 (5.93)
T_50_	Half-life to achieve half of the maximum effect	28.9 days (6.74)
HILL	Hill exponent in Sigmoid model	2.79 (9.59)
WGT_ON_CLQ	Weight on CL/Q	0.477 (12.1)
WGT_ON_VSS	Weight on V_ss_	0.970 (7.94)
ALT_ON_CLQ	ALT on CLQ	− 0.0795 (24.7)
ALB_ON_CLQ	ALB on CL/Q	− 0.926 (9.38)
IGG_ON_CLQ	IgG on CL/Q	0.184 (15.1)
BMI_ON_V_ss_	BMI on V_ss_	− 0.560 (15.3)
BLK_ON_T_50_	Black (race) on T_50_	1.01 (29.2)
IIV_CLQ	IIV on CL/Q	0.0870 (5.76)
IIV_V_ss_	IIV of V_ss_	0.0432 (6.44)
IIV_E_max_	IIV of E_max_	0.228 (15.5)
IIV_T_50_	IIV on T_50_	0.610 (17.2)
Ω	IIV between CLQ and V_ss_	0.0422 (8.47)

*ALB* albumin (g/L), *ALT* alanine aminotransferase (IU/L), *BMI* body mass index, *CL* clearance of cemiplimab in serum, *IIV* inter-individual variability, *IgG* immunoglobulin G (g/L), *Ω* variance–covariance matrix of the inter-individual random effects (η) in the PK or PD parameter, *PD* pharmacodynamics, *PopPK* population pharmacokinetics, *Q* inter-compartmental clearance between the central and peripheral compartments, *V*_ss_, volume of distribution

**Fig. 4:**
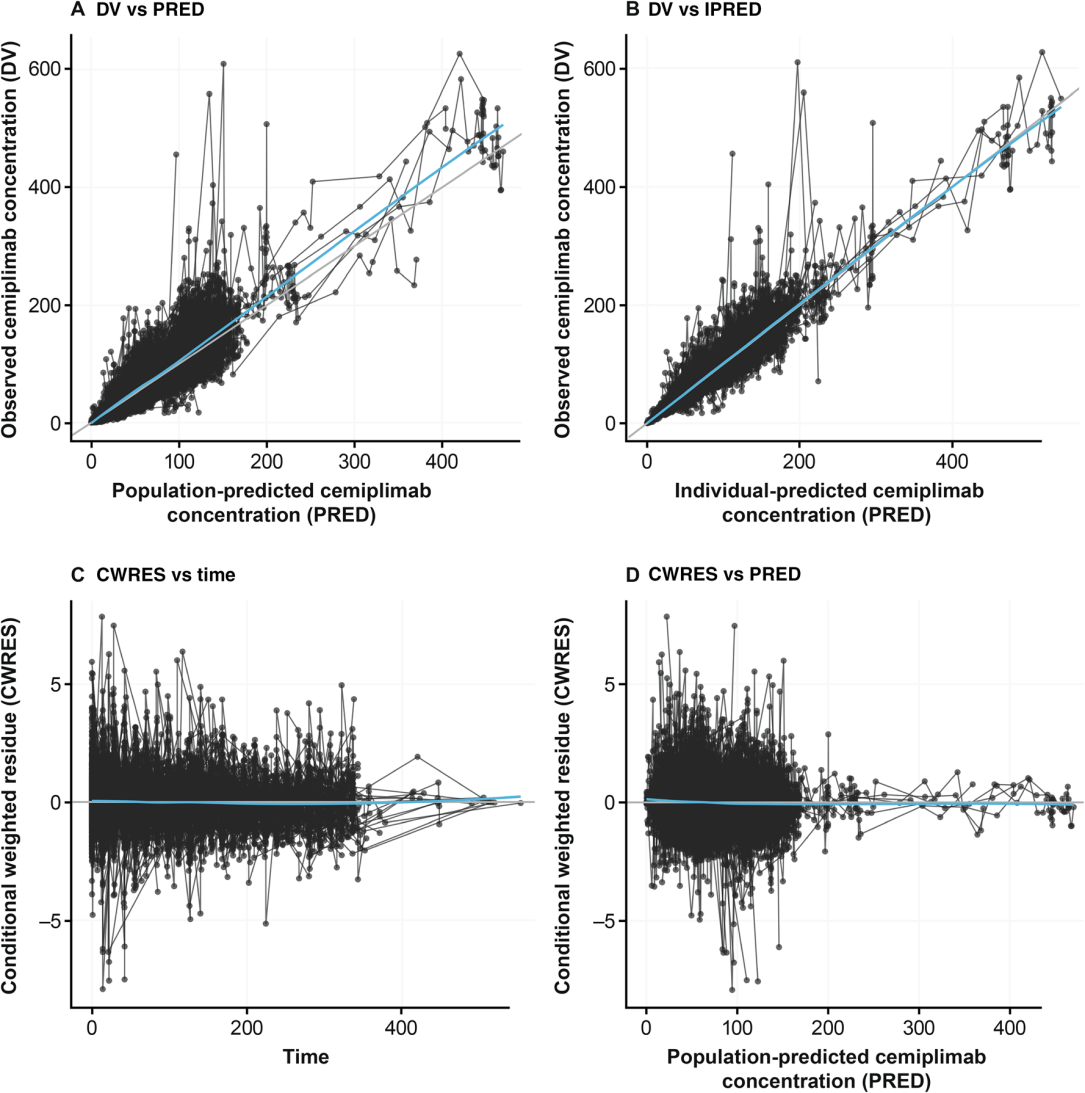
Diagnostic plots of final covariate model. **a**, **b** Observed (DV) vs population/individual-predicted (PRED/IPRED) cemiplimab concentrations. **c**, **d** Conditional weighted residue (CWRES) vs time and PRED

**Fig. 5:**
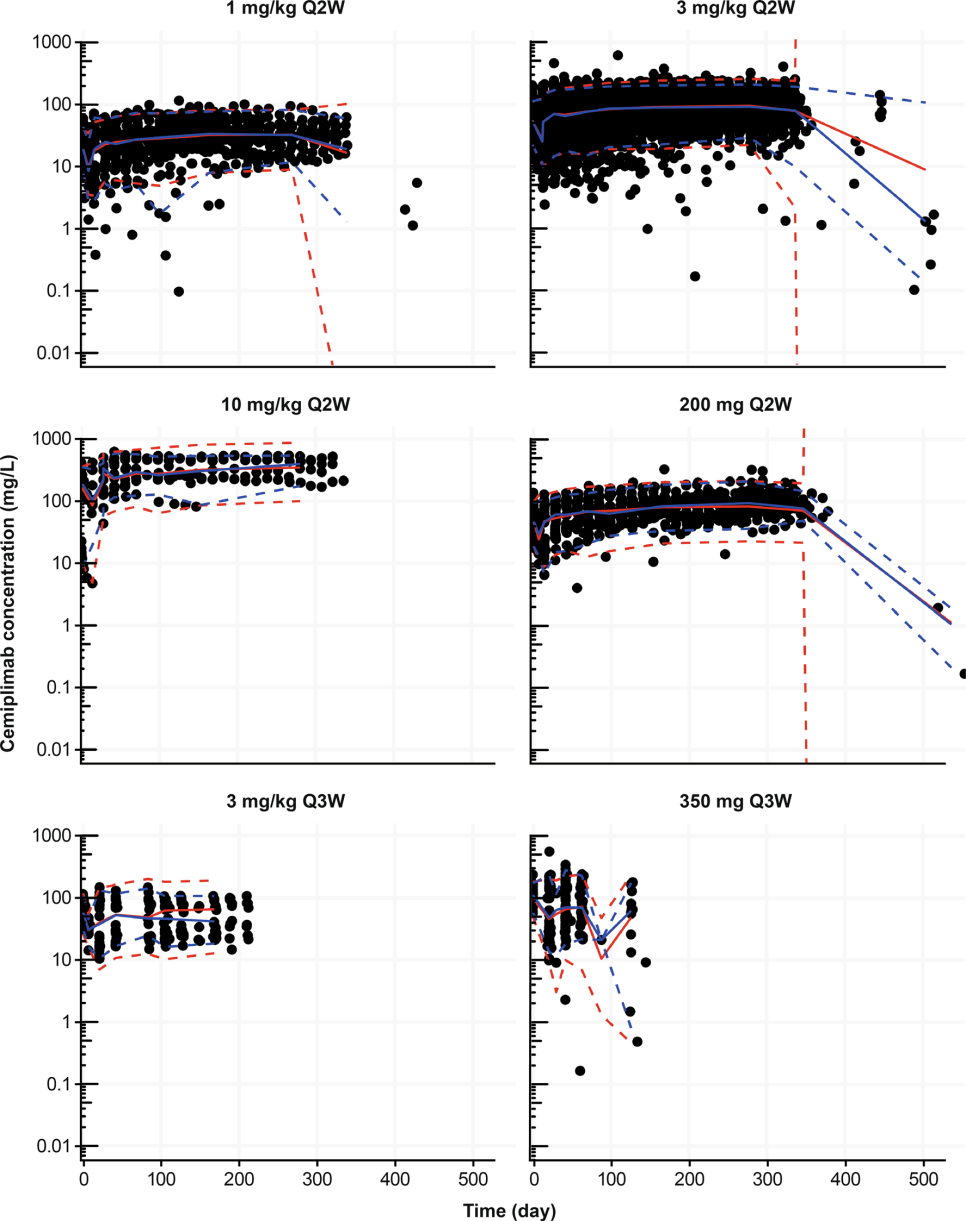
Visual predictive check plots for the PopPK model by dose groups in Studies 1423* and 1540. Black solid circles represent individually observed concentrations. Red and blue lines represent geometric mean (standard deviation) of individually predicted concentrations and geometric mean (standard deviation) of typical predicted concentrations, respectively. *One patient in the 10 mg/kg Q2W group of Study 1423 received the wrong dose (1 mg/kg) on Day 1; data from this patient are included in this figure. PopPK, population pharmacokinetics; Q2W, every 2 weeks; Q3W, every 3 weeks

Diagnostic (Fig. 4) and VPC (Fig. 5) plots showed good agreement between the observed data from clinical studies and predictions from the PopPK model.

### Simulated results informing Q3W fixed dose selection

**Fig. 6:**
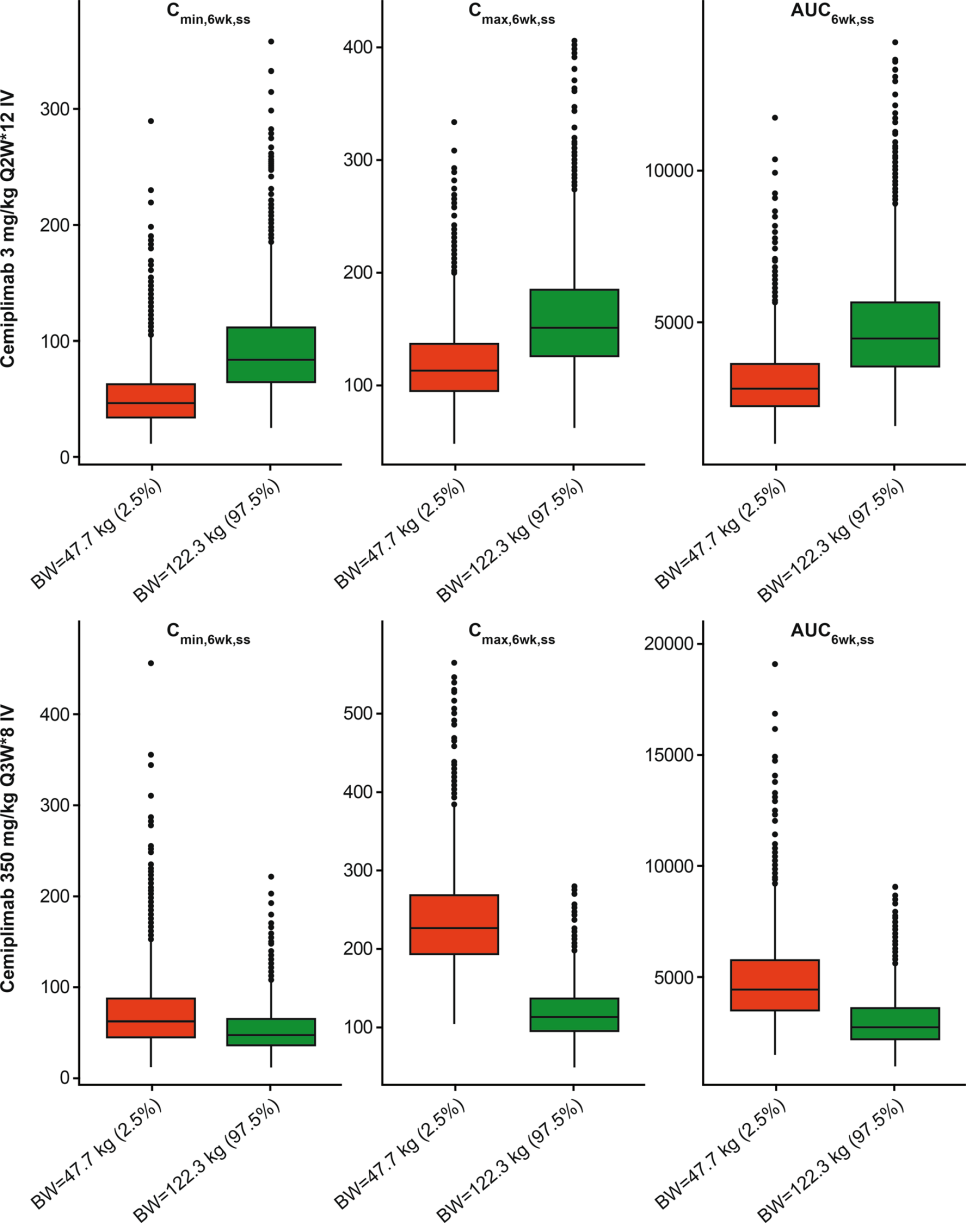
Boxplots of simulated cemiplimab exposure at steady state for BW extremes (N = 2000). AUC_6wk,ss_, area under curve over 6 weeks at steady state; BW, body weight; C_max,6wk,ss_, maximum concentration over 6 weeks at steady state; C_min,6wk,ss_, minimum concentration over 6 weeks at steady state; IV, intravenous; Q2W, every 2 weeks; Q3W, every 3 weeks

Simulations of cemiplimab exposure in 2000 typical patients with a median weight of 76.2 kg (range 30.9–172 kg; 2.5th percentile: 47.7 kg; 97.5th percentile: 122.3 kg) over a 24-week administration period showed similar variability in exposure for the 350 mg Q3W and 3 mg/kg Q2W dose regimens in patients with advanced malignancies at extreme weight (Fig. 6). Patients with higher body weight showed a trend towards higher cemiplimab exposure with the weight-based 3 mg/kg Q2W dose regimen; the trend was reversed for the fixed 350 mg Q3W dose regimen (Table 4). Both regimens produced similar overall exposure with similar variability across a wide range of body weights (30.9–172 kg).

**Table 4:** Post hoc estimates of AUC_6wk,ss_ and C_trough,ss_ by body weight quartiles at 3 mg/kg Q2W and 350 mg Q3W

Body weight quartiles (kg)	n	3 mg/kg Q2W		350 mg Q3W	
		AUC_6wk,ss_, mean (SD) (day mg/L)	C_trough,ss_, mean (SD) (mg/L)	AUC_6wk,ss_, mean (SD) (day mg/L)	C_trough,ss_, mean (SD) (mg/L)
Q1 (30.9, 65.5)	137	3280 (± 2220)	58.4 (± 51.6)	4510 (± 3180)	70.3 (± 72.6)
Q2 (65.5, 76.2)	138	3750 (± 1330)	66.8 (± 28.2)	4100 (± 1440)	64.0 (± 29.1)
Q3 (76.2, 88.5)	136	3960 (± 1220)	70.6 (± 26.3)	3760 (± 1190)	58.8 (± 24.5)
Q4 (88.5, 172)	137	4200 (± 1430)	74.0 (± 30.5)	3190 (± 1110)	49.0 (± 22.3)

*AUC*_6wk,ss_ area under curve over 6 weeks at steady state, *C*_trough,ss_, minimum concentration at steady state; *Q* quartile, *Q2W* every 2 weeks, *Q3W* every 3 weeks; *SD* standard deviation

### Observed cemiplimab serum concentration data supporting Q3W fixed dose selection

**Fig. 7:**
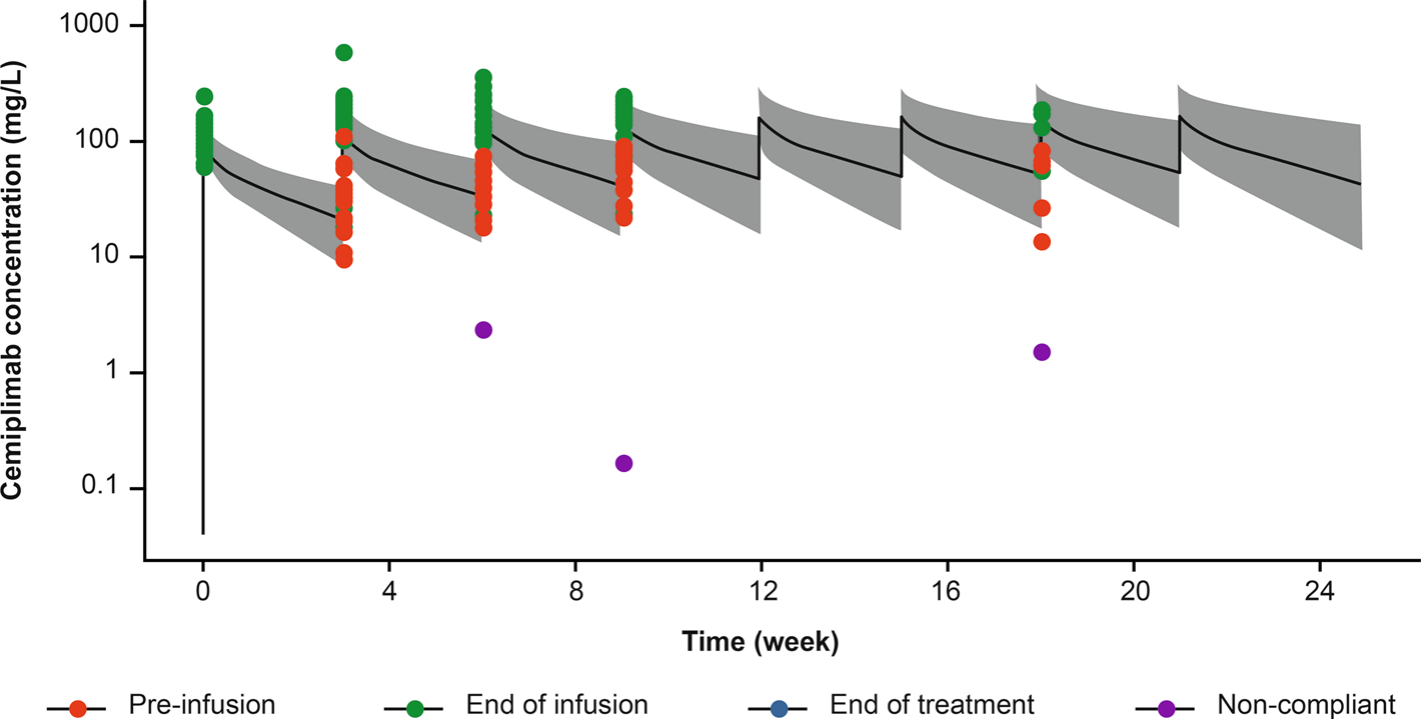
Overlay of observed and simulated cemiplimab concentration–time data at 350 mg Q3W. Plot shows the median (black line) and 95% CI (gray area) of simulated cemiplimab concentration–time data in 2000 patients with advanced malignancies overlaid with observed data points (dots) from 43 patients with advanced CSCC in Study 1540. Low pre-infusion concentrations were observed in two patients with missed doses (purple dots). CI, confidence interval; CSCC, cutaneous squamous cell carcinoma; Q3W, every 3 weeks

An overlay of observed and simulated concentration–time data at 350 mg Q3W showed comparable cemiplimab exposure for the same dose regimen (Fig. 7), confirming the PopPK simulation results. The plot showed that the observed concentration–time course of cemiplimab (2.5th to 97.5th percentiles) fell within the corresponding 95% prediction intervals, indicating that the model adequately predicted the central tendency (median) and extremes (2.5th and 97.5th percentiles) of the observed cemiplimab concentration–time data at 350 mg Q3W.

## Discussion

A two-compartment PopPK model with zero-order IV infusion rate, first-order elimination rate, and time-varying CL was found to well describe the concentrations of cemiplimab in patients. The simulation results from this PopPK model supported use of a fixed dose regimen of 350 mg Q3W as it provides comparable cemiplimab exposure to the initially studied weight-based dose regimen of 3 mg/kg Q2W. The PopPK results also supported approval of the fixed 350 mg Q3W dose regimen of cemiplimab by the US FDA (cemiplimab-rwlc) and the European Commission. Observed data from Study 1540 further confirmed this fixed dose selection [7].

### Linearity

As cemiplimab is a human monoclonal antibody directed against PD-1, a cell membrane target, data suggest that a saturable, target-mediated elimination pathway may be an important route of elimination at low doses and may lead to non-linear PK [5]. Indeed, such PK characteristics of cemiplimab were observed in cynomolgus monkeys during preclinical studies [1]. In the first-in-human Study 1423 with cemiplimab, dose-proportional, linear kinetics were observed at 1–10 mg/kg Q2W dose range after administration of the first dose over a 2-week dosing interval, indicative of systemic saturation of the underlying target-mediated pathways. In subsequent studies, a higher dose level (3 mg/kg Q2W) was selected to ensure maximum therapeutic effect at a systemic concentration sufficient to saturate the target-mediated pathway.

As the PK of cemiplimab were largely linear at the dose levels tested, a two-compartment linear PopPK model was found to adequately characterize the concentrations of cemiplimab in patients. These observations are consistent with the PK linearity observed with other anti–PD-(L)1 therapies (nivolumab, CT-011, and avelumab) over similar dose ranges [8–10]. Data from a single ascending dose and a multiple ascending dose study in patients with advanced solid malignancies showed that the PK of nivolumab was linear in the 0.1–10 mg/kg Q2W dose range with dose-proportional increase in maximum concentration and AUC and had low to moderate (20–44%) variability [8]. A Phase 1 study in patients with advanced hematologic malignancies demonstrated that the PK of CT-011 was linear after a single administration of 0.2–6.0 mg/kg [9]. In addition, PopPK analysis using data from patients with advanced solid tumors showed that a two-compartment model with linear elimination best described the PK of avelumab [10].

### Time-varying CL

CL of cemiplimab decreased by 35.9% over time compared with baseline CL (from 0.326 to 0.208 L/day within 16 weeks of treatment). However, this decrease in CL was not considered clinically meaningful. The time-varying CL of cemiplimab is consistent with observations in other anti–PD-1 therapies (nivolumab and pembrolizumab) [11, 12]. Time‐varying CL was demonstrated in a previous study of nivolumab using pooled data from multiple clinical trials [11]. Similarly, a study of pembrolizumab showed that pembrolizumab CL decreased over the treatment period of a typical patient with solid tumor in a pattern well described by a sigmoidal function of time [12]. The extent of decrease (35.9%) in cemiplimab CL from baseline over time was comparable to that observed with other anti–PD-(L)1 therapies (atezolizumab [17.1%] [13], pembrolizumab [21.0%] [12], durvalumab [22.9%] [14], nivolumab [24.5%] [11], and avelumab [41.7%] [15]).

In this study, although based on limited data, decrease in CL appeared to be more pronounced in treatment responders versus non-responders during cemiplimab treatment (Supplementary Fig. 1). Mean percent decrease in CL was larger in patients who responded to cemiplimab treatment versus those who did not respond (39.5% in responders vs 33.5% in non-responders). Consequently, patients with advanced CSCC who responded to cemiplimab treatment exhibited longer elimination half-life at steady state than other patients (mean 22.7 days vs mean 18.7 days). This interesting observation may be associated with the impact of disease state on time-varying CL of anti–PD-1 therapies. Nivolumab CL in patients with advanced melanoma was found to decrease during treatment, and the change in CL was associated with improved disease state, measured by reduced tumor burden [6, 11]. CL of pembrolizumab also decreased during treatment and was correlated to best overall response [12]. In a PopPK analysis of nivolumab administered as adjuvant therapy to patients with melanoma whose tumors were removed by surgical resection, who by definition had neither measurable tumor burden nor further-improved disease state, nivolumab CL was shown to be constant over time, further supporting the correlation between time-varying CL of anti–PD-1 therapies and tumor burden or disease state [16].

### Covariate analysis

Baseline body weight or BMI, albumin concentration, and IgG concentration were identified as the main sources of intrinsic variability for cemiplimab concentration in the PopPK analysis.

#### Albumin and IgG

The association between low albumin and increased CL in monoclonal antibodies is well documented and known to be related to the altered neonatal fragment crystallizable receptor-mediated recirculation of monoclonal antibodies [17]. However, the magnitude of increasing/decreasing cemiplimab exposure associated with low/high albumin levels was within the range of approximately 75–125% relative to the typical exposure at 350 mg Q3W. Considering cemiplimab is an IgG4 monoclonal antibody and the known physiological relationship of IgG proteins [18], endogenous or otherwise, with albumin [17], it is reasonable to expect the observed relationship between high baseline IgG levels and increasing cemiplimab CL in patients with advanced CSCC.

Other studies support the mechanism of albumin and IgG effects on PK. The results in this study are in agreement with a study in which extreme albumin levels were identified as a potential clinically meaningful predictor of vedolizumab CL in patients with ulcerative colitis and Crohn's disease (effect size > 25%) [19]. In addition, IgG has been shown to affect CL of daratumumab in patients with multiple myeloma, with approximately 110% higher CL in IgG myeloma patients than in non-IgG myeloma patients [20]. However, the effect of these covariates on cemiplimab exposure was not clinically significant (effect sizes ≤ 25% [19]).

#### Tumor type and burden

The data from this study did not indicate that clinically meaningful disease-related factors had an effect on cemiplimab concentration, including solid tumor types (CSCC [mCSCC or laCSCC] or other solid tumors) and baseline ECOG status. These observations are consistent with results from a PopPK analysis of pembrolizumab using pooled data from three clinical studies in patients with advanced melanoma, NSCLC, and other solid tumors. The pembrolizumab study showed that intrinsic factors (e.g., body weight, age, sex, tumor type and burden, and renal and hepatic impairment) and extrinsic factors (e.g., concomitant medications) had no clinically meaningful impact on pembrolizumab exposure [21].

#### Body weight

In this study, similar overall variability in cemiplimab exposure at 350 mg Q3W and 3 mg/kg Q2W was shown by PopPK modeling and simulations in patients with extreme body weights and across the full body weight range of 30.9–172 kg. Weight-based and fixed dose regimens of cemiplimab showed an opposite trend in change of exposure with body weight. The change of exposure relative to body weight was of a similar extent over a broad range of body weights, resulting in similar variability of exposure in a given patient population.

The variability had minimal impact on safety or clinical efficacy, considering that no dose-limiting toxicities were observed at a dose level as high as 10 mg/kg Q2W in Study 1423, clinical efficacy of cemiplimab was observed at a dose level as low as 1 mg/kg Q2W in Study 1423, and the exposure–response relationships are generally flat in anti–PD-1 therapies including cemiplimab over the concentration range studied [3, 22, 23].

#### Renal or hepatic impairment

Considering the molecular weight and hydrodynamic size of monoclonal antibodies, they are not subject to renal or hepatic elimination. Indeed, effects of baseline creatinine CL, creatinine concentration, and total bilirubin on cemiplimab exposure were small and not clinically meaningful (effect of covariate on cemiplimab concentration in serum < 20%), indicating that neither renal nor hepatic impairment had relevant effect on cemiplimab exposure. Similarly, a review of pembrolizumab and nivolumab showed that renal impairment and mild hepatic impairment had non-clinically significant effect on drug exposure and did not necessitate dosage adjustment [24].

### Exposure–response relationships

Anti–PD-1 therapies have shown relatively flat exposure–response relationships for both clinical efficacy and safety over a wide range of tested dosages, allowing flexibility in dose selection [22, 23]. This was demonstrated with nivolumab and pembrolizumab, suggesting that variability in exposure would not result in changes in clinical efficacy and/or safety measures. In subsequent studies of cemiplimab, the weight-based 3 mg/kg Q2W and the fixed 350 mg Q3W dose regimens demonstrated similar clinical efficacy and safety profiles [2, 25, 26].

### Summary

A robust PopPK model was developed to characterize the concentrations of cemiplimab in patients with advanced malignancies. Although several covariates, such as baseline body weight and albumin concentration, had a modest impact on the PK of cemiplimab, none was found to be clinically meaningful. Based on modeling and simulations using this PopPK model, a 350 mg fixed IV dose with a reduced dosing frequency (Q3W) was selected for further studies in patients with advanced malignancies, including advanced CSCC. These PopPK results also supported approval of the fixed 350 mg Q3W IV dose regimen of cemiplimab by the US FDA (cemiplimab-rwlc) and the European Commission. Similarity in observed cemiplimab exposure at the fixed 350 mg Q3W IV and at the weight-adjusted 3 mg/kg Q2W IV dose regimens further confirmed the 350 mg Q3W IV fixed dose selection.

## Supplementary Information

Below is the link to the Supplementary Information.

Supplementary Information 1 (DOCX 264 kb)
