# Supplementary material for: Population pharmacokinetic characteristics of cemiplimab in patients with advanced malignancies
Source: J Pharmacokinet Pharmacodyn. 2021 Mar 16;48(4):479–94. doi: 10.1007/s10928-021-09739-y (PMC8225544; doi:10.1007/s10928-021-09739-y)
Supplement: Supplementary file 1 — Supplementary Information 1 (DOCX 264 kb) [file 10928_2021_9739_MOESM1_ESM.docx]

Supplementary Fig. 1. Post hoc individual estimates of cemiplimab clearance over time by treatment response in patients with CSCC


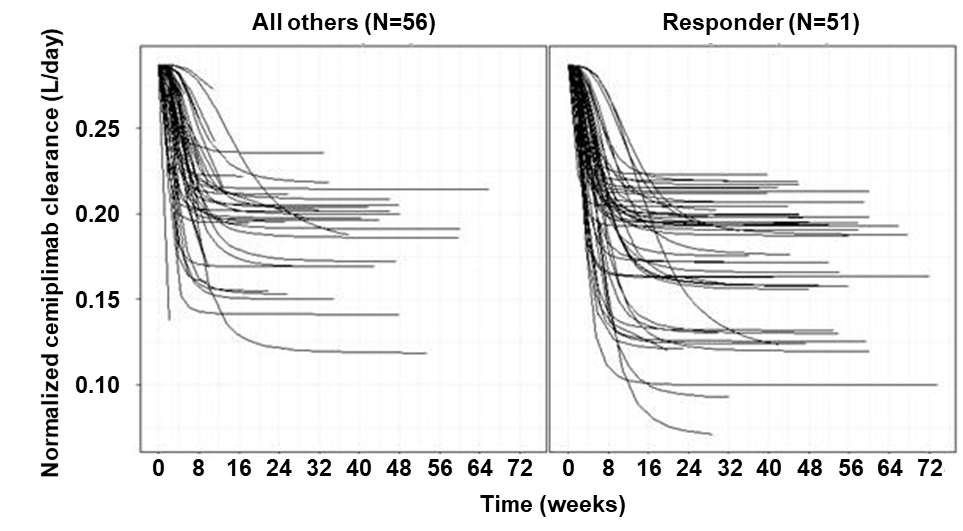


Plot shows normalized cemiplimab clearance over time from a total of 107 patients with CSCC (51 responders; 56 non-responders) in the clinical efficacy analysis population. One responder received 1 mg/kg Q2W cemiplimab; all other responders received 3 mg/kg Q2W cemiplimab.

CSCC, cutaneous squamous cell carcinoma; Q2W, every 2 weeks.
